# Layman versus Professional Musician: Who Makes the Better Judge?

**DOI:** 10.1371/journal.pone.0135394

**Published:** 2015-08-26

**Authors:** Pauline Larrouy-Maestri, David Magis, Matthias Grabenhorst, Dominique Morsomme

**Affiliations:** 1 Neuroscience Department, Max-Planck-Institute for Empirical Aesthetics, Frankfurt, Germany; 2 Fonds de la Recherche Scientifique-FNRS, Brussels, Belgium; 3 Psychology Department, University of Liège, Liège, Belgium; Max Planck Institute for Human Cognitive and Brain Sciences, GERMANY

## Abstract

The increasing number of casting shows and talent contests in the media over the past years suggests a public interest in rating the quality of vocal performances. In many of these formats, laymen alongside music experts act as judges. Whereas experts' judgments are considered objective and reliable when it comes to evaluating singing voice, little is known about laymen’s ability to evaluate peers. On the one hand, layman listeners–who by definition did not have any formal training or regular musical practice–are known to have internalized the musical rules on which singing accuracy is based. On the other hand, layman listeners’ judgment of their own vocal skills is highly inaccurate. Also, when compared with that of music experts, their level of competence in pitch perception has proven limited. The present study investigates laypersons' ability to objectively evaluate melodies performed by untrained singers. For this purpose, laymen listeners were asked to judge sung melodies. The results were compared with those of music experts who had performed the same task in a previous study. Interestingly, the findings show a high objectivity and reliability in layman listeners. Whereas both the laymen's and experts' definition of pitch accuracy overlap, differences regarding the musical criteria employed in the rating task were evident. The findings suggest that the effect of expertise is circumscribed and limited and supports the view that laypersons make trustworthy judges when evaluating the pitch accuracy of untrained singers.

## Introduction

Although television casting shows are indeed appealing to the general audience, expert music listeners more often than not explicitly reject such formats. This is not surprising, considering the general assumption that music experts knowingly employ specific criteria to describe the quality of a vocal performance whereas laypersons–who may be avid music listeners–are deemed ignorant of these criteria and their appropriate use. However, it is unclear whether there are differences in the way audiences comprised of either experts or layman listeners evaluate and appreciate the performance of sung melody. This paper examines the way listeners evaluate vocal performance, with a focus on pitch accuracy (a critical parameter in defining singing talent [1]), and investigates the potential effects of formal training on the perception of singing voices.

### Evaluation of pitch accuracy in melodies

A melody is a succession of tones following conventions and constraints dictated by a musical system specific to a culture [2–4]. In this context, singing “in tune” is commonly defined as performing in congruence with these rules.

In the musical system of the Western culture, three kinds of melodic errors can be observed: 1) incorrect melodic contour (e.g., performing an ascending interval instead of a descending one), 2) incorrect interval size between two tones, and 3) unintended modulation (i.e., change in tonality). These errors can be objectively quantified by computer-assisted methods which extract the fundamental frequency (F0) of each tone and compare their relationships with the expected ones (see [5] for a review of analytical tools and procedures). In addition to avoiding the influence of subjectivity, this process can be used to identify criteria that listeners employ when listening to sung melodies. As an example, we have recently investigated the relevance of the three aforementioned criteria (i.e., contour, interval size and tonality of a melody) in evaluating performances of occasional singers [6]. In this study, interval size and tonality explained 81% of the variance of the experts in voice/music judges’ ratings, whereas contour errors did not appear as relevant. In other words, listeners seem to pay particular attention to these two musical criteria when listening to ecological material. Our previous findings demonstrate a high degree of objectivity in music experts' evaluation of sung performances. Another quality marker in music evaluation is the intra- and inter-judges agreement. Our previous findings regarding the evaluation of pitch accuracy confirm the reliability of judges, as already pointed out by Wise and Sloboda [7] and Racette, Bard, and Peretz [8]. In the evaluation of occasional singers, we observed that a small group of expert judges (around 3) is enough to keep a strong relationship between the mean rating and the objective measurements [6]. However, the consistency and objectivity highlighted in previous studies is limited to audiences consisting of music experts.

To our knowledge, the ability of layman listeners to judge layman singers (i.e., who only sing occasionally and do not have formal musical training, also called “occasional singers”) has only been investigated in the context of self-evaluation. In such context, the participants show difficulties in evaluating their own singing proficiency. For instance, Pfordresher and Brown [9] report that in a sample of 1105 students, 59% claimed to be unable to imitate a simple melody, while 15% had difficulty to accurately imitate melodic sequences. This finding underpins the difficulty of self-evaluation which most people experience [7, 10–12] and which is found in many different domains (e.g., [13, 14]). Additionally, Pfordresher and Brown’s findings could reflect a general difficulty of non-musicians to accurately evaluate the precision of melodies performed by layman singers. In order to clarify whether the reasons for such difficulty are related to the self-evaluation process or if they reflect a fundamental difficulty to evaluate sung performances, this study focuses on the layman's ability to carry out an objective and reliable evaluation of another layman's performance.

### Music experts versus laymen listeners

An expert is commonly defined as somebody who acquired special skills or knowledge of a particular subject through training and practical experience. Therefore, musical expertise is often associated with a great amount [15–19] and also with good quality [20, 21]) of deliberate music practice. More recently, the debate about the nurture/nature relative influence has been nourished by genetic evidence [22, 23], which highlights the complexity of both origin and development of musical expertise [24–26]. Since the definition of musical expertise and the factors influencing its development have yet to be clarified, an alternative view may define music experts as individuals who reach a high level of musical performance skills [27] and fulfill several criteria, such as playing music as the main source of income or the recognition by peers or audience [28]. Music expertise cannot only be acquired through formal musical training but the type and level of formal musical practice by an individual is generally considered a standard measurement of their expertise.

The literature on the effects of music expertise is vast and we limit ourselves here to musical skills relevant in evaluation of pitch accuracy. Regarding the discrimination of pure tones, Moore [29] reports that trained musicians are able to distinguish pure tones with an accuracy of 0.2% at 1kHz. When compared to non-musicians, trained musicians show better discrimination abilities for pure tones and complex sounds [30, 31]. In addition to the superior performances of musicians in discrimination tasks ([32] for a review), they also excel on pitch perception tasks with isolated tones [33]. Musicians also outperform non-musicians in estimating the size of musical intervals [34], when comparing complex sounds (vocal or instrumental) of different timbres in the context of isolated tones [35, 36] or intervals [37]. In melodic contexts, they detect pitch deviations with better precision [35, 38], they are better in identifying changes in contour and interval [39] and their pitch processing is more effective [40]. Musicians integrate tonal structure better than non-musicians [41], their processing of melodic material is faster [30, 42, 43], and their temporal integration window is more precise [44]. Note that when rating musical performances, some authors observed that the inter-judges’ reliability increases with the expertise of the judges [45, 46]. Previous studies did not find this effect [47–50], which could be explained by a lack of control regarding the type/level of musical expertise.

The numerous differences between musicians and non-musicians reported in the aforementioned studies support the hypothesis that the mental representations of melodies and therefore the definition of pitch accuracy would be less precise in non-musicians, leading to less objective and less consistent ratings. However, several points can be made to support the claim that layman listeners are also qualified judges.

First, among the several studies contrasting music experts and non-experts, some reported similar performances of the two groups, especially on tasks described as “simple”. For instance, Besson and Faïta [51] observed better performances in music experts compared with non-experts in a musical incongruity detection task but did not observe any difference if the incongruities were easily detectable. Since the evaluation of singing voice is an immensely popular task, as illustrated by the myriad of casting formats and singing talent contests, rating sung performances should not be considered difficult per se.

Second, we are all exposed to the music of our specific culture and are able to implicitly learn a system of musical rules [52, 53]. Similar to language acquisition, musical enculturation shapes perceptual abilities: a child does not need specific training to become an “expert listener” in his or her culture [54, 55] (see also Müllensiefen et.al [56] for a discussion on this topic). According to Stalinski and Schellenberg, the enculturation process ends around the age of 5 years [57]. Thus, even young children acquire musical knowledge, which allows them to understand musical structure [58, 59], and to develop melodic expectations [60]. In addition to being naturally acquainted with the “vocal instrument” (i.e., informal training in speaking and singing), young children also develop sensitivity to melodic errors such as violation of melodic contour [61, 62], deviation of the interval size [63] and changes in tonality [64] found in musical material. Therefore, despite an absence of formal training in music, non-musicians are sensitive to the musical rules of their culture, to the timbre of the vocal instrument itself, which qualifies layman listeners as “experienced listeners” (see [65] for review).

Nevertheless, the fact that rating melodies can be viewed as a simple task and that layman listeners are experts in their own culture, does not mean that they actually share a similar definition of pitch accuracy and use similar rating strategies. This study aims (i) to clarify how layman listeners define pitch accuracy and (ii), by means of comparison with experts, to examine the consistency and objectivity of layman listeners when evaluating “simple” sung performances.

## Methods

We applied the procedure described in Larrouy-Maestri et al. [6] to layman listeners (see Participants section below). In this reference study, participants were a group of 18 experts (8 women) aged from 19 to 51 years old (*M* = 33.33, *SD* = 9.87), with formal training in music or singing voice: Professional musicians, highly trained music students, vocal coaches, and singers (for further details, see [6]). They were asked to rate 166 performances (http://sldr.org/sldr000774/en) of the song "Happy Birthday" (with French lyrics), performed a cappella by 109 women and 57 men (14–76 years old, *M* = 29.89 years), on a 9-point scale, from very inaccurate to very accurate. There was no difference between subgroups within the group of experts, irrespective of the different kinds of formal training. Each performance was previously analyzed regarding pitch interval deviation, number of contour errors and tonality modulations (Table 1).

**Table 1:** Description (Mean, Standard Deviation, Minimum, Maximum) of the three criteria analyzed in the 166 melodic performances. The 166 performances were analyzed with AudioSculpt 2.9.4v3 and OpenMusic 6.3 software (IRCAM, Paris, France) using a Short Time Fourier Transform (STFT), with regard to equal temperament. For an extensive description of the analytical procedure of pitch accuracy see [5]. The pitch interval deviation criterion represents the mean absolute value of the differences between the performed intervals and the theoretical ones along each melody. A contour error is counted when the produced interval is in the opposite direction of the expected one (i.e., ascending/descending). A tonality modulation corresponds to an interval error larger than a semitone not followed by a corrective interval of at least a semitone in the reverse direction.

Musical criteria	Mean (SD)	Minimum	Maximum
Pitch interval deviation (cents)	55.97 (48.21)	10.10	383
Number of contour errors	0.14 (0.66)	0	5
Number of tonality modulations	1.89 (1.98)	0	8

### Ethics statement

Informed signed consent was obtained from each participant in accordance with the human subjects’ research protocol approved by the Ethics Committee of the Psychology Department of the University of Liège (Belgium).

### Participants

Eighteen layman listeners (*M* = 33.06 years old, *SD* = 9.57) were paired in gender (8 women) and in age (*t*(34) = .278, *p* = .93) with the expert listeners of the reference study [6]. They were recruited in Belgium and France. The following inclusion criteria were applied: (a) bilateral hearing threshold of 20 dB SPL at 500, 1000, 2000, and 4000 Hz, screened with pure tone audiometry (Madsen Xeta, GN Otometrics, Denmark); (b) no history of choral singing and no history of formal musical training (or maximum 2 years of musical training and no practice during the past 5 years); (c) no congenital amusia (tested with the Montreal Battery of Evaluation of Amusia, MBEA [66], (d) no particular appetence to music (attending less than one concert a week and actively listening to music less than two hours a day), and (e) the ability to perform the song Happy Birthday with respect to appropriate melodic contour. Note that none of them mentioned possessing absolute pitch.

### Procedure

Like the expert judges of the reference study, the layman listeners were asked to listen to the 166 samples via headphones (K271 MKII, AKG, Vienna, Austria) and to rate each sample on a 9-point-scale, from 1 “very inaccurate” to 9 “very accurate”. Five randomized lists were proposed and four trials were presented prior to the rating task. The procedure was repeated after 8 to 15 days (*M* = 9.44 days).

### Statistical analyses

Three successive analyses were performed.

In **Analysis 1**, nine figures containing several boxplots were created, depicting all possible combinations of judges within one group (non-experts at test, non-experts at retest, experts) and the explanatory variables objectively analyzed (pitch interval deviation, number of contour errors, and number of modulations). Each figure was drawn as follows. First, boxplots were produced for each possible size of subsets of judges (from one judge to all 18 judges). Second, for each given number of judges (i.e. n), all possible subsets of n judges among the 18 were considered and the average score of all samples was computed for each subset of judges, leading to one average score per sample (the average depending on the selected judges). Finally, Spearman correlations between the average scores and the selected explanatory variable were computed, leading to one correlation per selected subset of judges. These Spearman correlations were eventually displayed as boxplots, leading to 18 boxplots per figure, each boxplot referring to a particular number of selected judges (from 1 to 18) and displaying all correlations between the selected variable and the mean scores (from one of the three sets of scores) of the subsets of judges.

In **Analysis 2**, non-expert judges' scores were analyzed with respect to three explanatory variables (pitch interval deviation, number of contour errors, and number of modulations, see Table 1) in a regular linear model. A single score was assigned to each performance computed as the median score across all non-expert judges. Significant effects of explanatory variables were assessed by t-tests.

**Analysis 3** compares the layman listeners to the experts group. A repeated-measurements linear model was built to analyze the effect of the same three explanatory variables (pitch interval deviation, number of contour errors, and number of modulations) on median scores of non-expert and expert judges. Repeated measures were set between non-expert and expert judges' scores as they were obtained using the same set of samples. The effect of each explanatory variable was first modeled separately for each subset of judges (expert and non-expert), then tested by means of usual statistical significance tests. The simplest model without non-significant terms was eventually retained for analysis and discussion.

All statistical analyses were performed with the R software (R Core Team, 2014). Throughout the analyses the significance level was fixed to 5%.

## Results and Discussion

**Fig 1:**
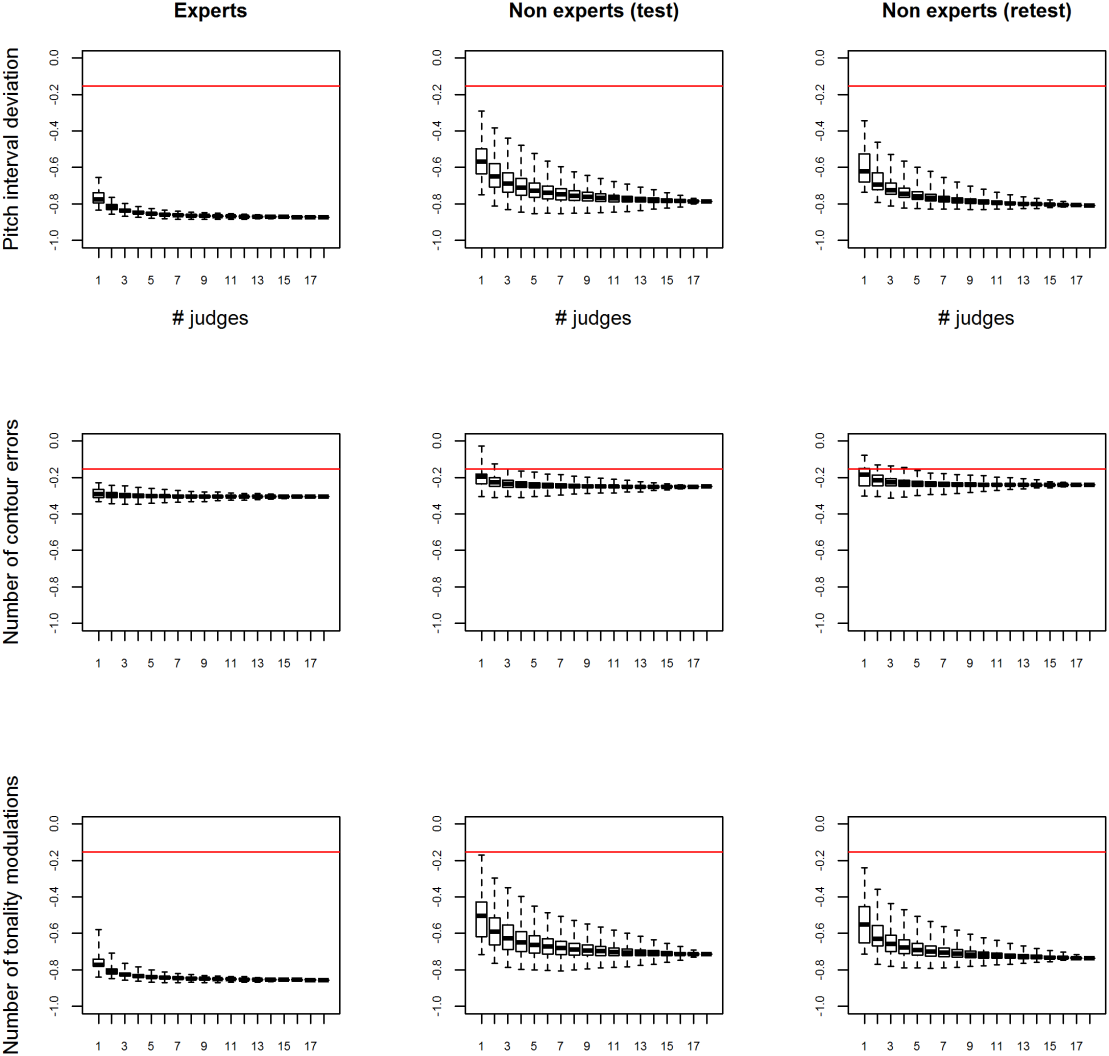
Relationships between judges’ ratings and musical criteria. Boxplots of Spearman correlations between either pitch interval deviation (top), number of contour errors (middle) or number of modulations (bottom), and the average scores of expert judges (left), non-expert judges at the test (middle) and non-expert judges at the retest (right). Each boxplot refers to a particular number of judges and all possible combinations of judges are considered. Horizontal lines indicate the threshold for significant correlation coefficients (based on samples of 166 performances at significance level 5%).

Fig 1 shows the results of **Analysis 1**, depicting the relationships between the judges’ ratings and the three musical criteria under study (pitch interval deviation, number of contour errors, and number of modulations).

For two of the three musical criteria, i.e. pitch interval deviation and number of modulations, the median correlations with the average score given by the judges were high (higher scores for accurate performances) and highly significant. Note that in the case of the expert judges in the reference study, a group size of only three resulted in a correlation of about .83 between their scores and the pitch interval deviation measurement, and .81 between their scores and the number of modulations. Regarding the non-experts, we also observed a strong relationship between the average scores and the two criteria (about .79 for the pitch interval deviation and .71 for the number of modulations). However, the variability in the non-experts' judgments is visibly larger (as can be seen by the width of the whiskers in Fig 1) compared to the one of the experts, especially with a small number of judges in the sample. This finding confirms that expertise enhances inter-judges reliability [45, 46]. In addition, this variability was lower at retest. So inter-judges reliability in the non-experts group was improved at the time of the second evaluation. Therefore, even a short training (i.e., a previous session 8 to 15 days before) seems to impact the definition of pitch accuracy in layman listeners. However, the median correlation is always smaller (absolute value) than in the experts group, even when considering the full sample of judges (n = 18) and independent of the time of evaluation (i.e., test or retest). In other words, the objectivity of layman listeners on the one hand, reflects adequate learning [54, 55, 60] and the use of implicit learning of musical rules [57–59, 61, 62, 64]. On the other hand, the objectivity seems less pronounced than the experts’ one and does not seem to increase at retest (unlike the variability). Further investigation, with repeated sessions would allow for clarifying the effect of short-term training (e.g., realizing the task several times with/without feedback on the accuracy of rating) on variability and objectivity of layman listeners. Note that among the three musical criteria objectively analyzed, the correlation between the number of contour errors and the average score given by the non-expert judges was significant but particularly low (*r*(18) = 0.24, *p* < .05), irrespective of the time point of evaluation (test versus retest). As illustrated in Fig 1, this pattern of results is similar to the one of the expert judges of the reference study (*r*(18) = 0.3, *p* < .05), and could be explained by the small number of errors of this kind (see Table 1) in the database (*n* = 166 untrained singers from the general population) due to the familiarity of the chosen melody.

**Analysis 2** revealed that the effect of pitch interval deviation on non-expert judges' scores was highly significant, while the effects of number of contour errors and the number of modulations did not reach statistical significance (Table 2). The R-squared coefficient for this model was .665. As can be seen in Table 2, R-squared coefficient in the case of music experts of the reference study was about .81.

**Table 2:** Summary of the multiple regression analysis on non-experts’ scores with the three musical criteria (pitch interval deviation, number of contour errors, and number of tonality modulations) used as predictors. For each variable, the beta weights and significance tests are represented. The columns on the right summarize the results of a similar analysis with group of music experts from Larrouy-Maestri et al. [6].

	Non-experts: *R* ^*2*^ = .665			Experts: *R* ^*2*^ = .81		
	*beta*	*t*	*p*	*beta*	*t*	*p*
Pitch interval deviation	-0.96	-7.04	< 0.001	-0.51	-6.61	< 0.001
# contour errors	-0.01	-0.19	0.85	.08	1.89	.06
# modulations	-0.14	-1.08	0.28	-.45	-6.32	< 0.001

It can be concluded that only the pitch interval deviation variable has an impact on the median scores of the non-expert judges: Larger deviations of pitch intervals lead to lower scores. This analysis confirms the objectivity of the layman listeners when evaluating melodies performed by occasional singers. It supports the hypothesis that listeners’ previous exposure to music allows for the internalization of musical rules [2–4], in particular those that apply to interval size, and more importantly, displays laypersons' ability to use these rules in ecological settings. The mechanisms for this kind of internalization of rules may be closely related to action-perception coupling. In the context of music, action-perception coupling refers to the coupling of motor and auditory cortices due to recurrent performance of a sensorimotor task [67] and has been observed not only in proficient players of various musical instruments [68–70], but also in naive participants who only received short musical training [71]. As it is very likely that the participants have themselves sung the song "Happy Birthday" numerous times, the concept of action-perception coupling could add to understanding the mechanisms of musical rule internalization found in our participants.

In the present study, the participants were not asked to evaluate their own performances. A direct comparison with previous studies on self-reports [7, 9–12] would therefore be inadequate. However, our results support that the difficulties of layman listeners in correctly evaluating their own performances cannot be attributed to a general difficulty of non-musicians in evaluating the accuracy of sung performances. This is in line with studies from other domains, which show that even experts have difficulty in self-evaluation (e.g., [14, 72]). Interestingly, this analysis shows that the definition of pitch accuracy does not include the number of tonality modulations, a finding that is in stark contrast to the music experts in the reference study. Adult listeners are able to perceive tonal violations [64] but this ability appears later in development, after the integration of information relative to musical intervals [73]. Since tonal deviations are obviously perceived by layman listeners (strong relationship between the number of modulations and the judges’ rating), implicit learning of musical rules is perhaps not sufficient to “apply” this musical criterion to the evaluation of melodic performances.

In a broader sense, this finding exemplifies the difficulty to distinguish between musical expertise and musical education. Several different approaches are commonly employed to describe musical expertise (see above), reflecting that proficiency in music has numerous facets. However, an intense formal musical education does neither guarantee a high level of expertise in music performance or evaluation, nor proves necessary when it comes to achieving musical competence. Recent literature shows the progress that has been made to incorporate this diversity and also strives to more accurately describe musical competences (see [56] for a review). However, categorization of competence in singing and evaluation of singing voices is not a simple endeavor [74], a notion the results of the present paper support. In light of the many possible facets and definitions of musical expertise, a valid musical skills test or questionnaire that does not entirely rely on the commonly employed criteria (music education, music as a professional activity) would be a highly desirable tool in this line of research.

The benefits of formal training are supported by **Analysis 3**, which consisted of a statistical comparison of previously acquired data [6] and present data. This analysis showed that the variable relative to pitch interval deviation has a significant effect on judges' scores (coefficient = -3.432, *t* = -7.328, *p* < 0.001), but this effect is the same across groups (non-expert and expert) of judges (coefficient = 0.274, *t* = 0.324, *p* = 0.746). Note that the effect of the pitch interval deviation variable is similar to that of **Analysis 2**: Larger pitch interval deviation leads to lower judges' scores. Also, there exists a significant effect of the variable relative to the number of modulations that differs across groups of judges. More precisely, the effect of number of modulations is not significant (*F* = 1.275, df = 1, *p* = 0.295) for non-expert judges, while for expert judges larger number of modulations lead to lower scores (coefficient = -0.459, *t* = -6.559, *p* <0.001). In other words, the layman listeners' definition of pitch accuracy is mainly based on the size of the intervals along a melody. Note that rating melodies containing a greater number of contour or modulation errors may lead to a different pattern of results. Indeed, greater variability along one dimension (i.e., pitch interval deviation) may draw the judges’ attention to this specific dimension. However, the median correlations found between the judges’ ratings and the three music criteria under study support that the differences in variability cannot fully explain the result of Analysis 2. In addition, adding contour or modulation errors would generate material which would not be as representative of the singing ability of the general population as the material used in the present study. Surprisingly, **Analysis 3** also shows an overall effect of the subgroup of judges on the magnitude of the rating. Non-experts were on average more “strict” and returning lower scores than expert judges (coefficient = -0.634, *t* = -4.465, *p* < 0.001). In view of the multiple benefits of formal music expertise on discrimination abilities ([32] see for review) and their consequences on pitch processing [33–40], the opposite results were expected. Two possible explanations can be proposed. First, music experts are used to evaluating music performances of trained musicians and therefore may be more tolerant concerning flaws in pitch accuracy of untrained singers. It may be that the non-experts expect better quality of peer performances due to their reference to recorded material (i.e., popular music which is produced with very limited tolerances for pitch imperfections). Second, the music experts and non-experts may be similarly objective and tolerant (similar correlation coefficients between objective measures and judges’ ratings), but they show a different use of notation scales. Future studies comparing different rating tools (forced-choice versus pairwise comparisons) in music experts and non-experts would provide additional arguments to explain this difference in rating magnitude.

## Conclusion and Perspectives

By replicating a previous study on music experts [6], but using non-experts instead, we examined the ability of layman listeners to evaluate familiar melodies performed by laymen (i.e., occasional singers). Taken together, the results highlight the objectivity and relative reliability of listeners without formal music training in evaluating melodies performed by occasional singers. However, these results raise several new questions. If layman listeners are capable of objectively evaluating sung performances of familiar melodies performed by their peers, it does not mean that they are “experts” per se. More likely, their ability is rather similar to that shown by experts in the context of evaluating popular tonal melodies with simple musical rules performed with the vocal instrument. In order to further investigate whether musical perception does benefit from formal musical expertise, a design using familiar melodies and either atonal material, complex acoustical signals such as operatic voices [75], or complex musical structures, or foreign musical rules could be proposed. In addition, the effect of expertise (shown elsewhere) may not directly affect the definition of pitch accuracy itself but rather the evaluation process. In other words, experts are more used to function as judges of musical performance. This hypothesis is supported by the non-experts' larger variability in rating and their higher strictness of judgment (i.e., low global score). The latter fact may be explained by an actual greater variance in tolerance thresholds among layman listeners [76]. The observed larger variability in ratings in the non-experts group again lends support to this hypothesis. Finally, although the definition of pitch accuracy in melodic contexts seems not to be strongly influenced by the quality of the signal or other musical criteria, as evidenced by the high percentage of variance that is explained, the quality of the voice (e.g., jitter, shimmer, signal noise ratio), the rhythmic component, and the scoops at the start and end of tones may have an impact on the evaluation process of pitch accuracy. These parameters, as well as the number of errors contained in the material, may be manipulated in future studies by using synthesized musical material to examine their influence on the rating process.

Despite the limitations of natural stimulus material, our study clearly shows the ability of layman listeners to evaluate pitch accuracy in the context of ecological melodic performances. By extension, the design of the studies presented here could also facilitate the investigation of the influence of visual cues (e.g. [77]), other musical timbre [35, 36], or more subjective aspects of music performance perception. For instance, the methods used to examine the evaluation of pitch accuracy as a technical component of singing could easily be adapted to examine more general aspects, such as music preferences among musicophiles and non-musicophiles and would thus contribute to a better understanding of music perception and appreciation.

## References

[1] Watts C, Barnes-Burroughs K, Andrianopoulos M, Carr M. Potential factors related to untrained singing talent: a survey of singing pedagogues. J Voice. 2003;17(3):298–307. 14513953 10.1067/s0892-1997(03)00068-7

[2] Cross I. Music, Cognition, Culture, and Evolution. Ann N Y Acad Sci. 2001;930:28–42. 11458835 10.1111/j.1749-6632.2001.tb05723.x

[3] Lerdahl F, Jackendoff R. A generative theory of tonal music. Cambridge, MA: MIT Press; 1983.

[4] Ringer AL. Melody: Definition and origins. Macmillan Online Publishing; 2002 Available: http://www.grovemusic.com.

[5] Larrouy-Maestri P, Morsomme D. Criteria and tools for objectively analysing the vocal accuracy of a popular song. Logoped Phoniatr Vocol. 2014;39(1):11–18. 10.3109/14015439.2012.696139 22721558

[6] Larrouy-Maestri P, Lévêque Y, Schön D, Giovanni A, Morsomme D. The evaluation of singing voice accuracy: A comparison between subjective and objective methods. J Voice. 2013;27(2):259.e1–259.e5. 10.1016/j.jvoice.2012.11.003 23280380

[7] Wise KJ, Sloboda JA. Establishing an empirical profile of self-defined "tone deafness": Perception, singing performance and self-assessment. Music Sci. 2008;12(1):3–26.

[8] Racette A, Bard C, Peretz I. Making non-fluent aphasics speak: sing along! Brain. 2006;129(10):2571–2584. 16959816 10.1093/brain/awl250

[9] Pfordresher PQ, Brown S. Poor-Pitch Singing in the Absence of "Tone Deafness". Music Percept. 2007;25(2):95–115.

[10] Cuddy LL, Balkwill LL, Peretz I, Holden RR. Musical difficulties are rare: a study of "tone deafness" among university students. Ann N Y Acad Sci. 2005;1060:311–324. 16597781 10.1196/annals.1360.026

[11] Sloboda JA, Wise KJ, Peretz I. Quantifying tone deafness in the general population. Ann N Y Acad Sci. 2005;1060:255–261. 16597772 10.1196/annals.1360.018

[12] Wise KJ, Sloboda JA, Peretz I. Progress in Understanding "Tone Deafness". Brit Acad Rev. 2007(10):52–54.

[13] Burson KA, Larrick RP, Klayman J. Skilled or unskilled, but still unaware of it: How perceptions of difficulty drive miscalibration in relative comparisons. J Pers Soc Psychol. 2006;90(1):60–77. 16448310 10.1037/0022-3514.90.1.60

[14] Parker ZJ, Waller G. Factors related to psychotherapists' self-assessment when treating anxiety and other disorders. Behav Res Ther. 2015;66:1–7. 10.1016/j.brat.2014.12.010 25614972

[15] Ackerman PL. Nonsense, common sense, and science of expert performance: Talent and individual differences. Intelligence. 2014;45:6–17.

[16] Ericsson KE, Krampe RT, Tesch-Römer C. The role of deliberate practice in the acquisition of expert performance. APA. 1993;100:363–406.

[17] Lehmann AC, Ericsson KA. Research on experts performance and deliberate practice: implications for the education of amateur musicians and music students. Psychomusicology. 1997;16:40–58.

[18] Lehmann AC, Gruber H. Music In: Ericsson K.A C N, Feltovich P.J., Hoffman R.R., editor. The Cambridge Handbook of Expertise and Expert Performances. 2006 p. 457–470.

[19] Platz F, Kopiez R, Lehmann AC, Wolf A. The influence of deliberate practice on musical achievement: a meta-analysis. Front Psychol. 2014;5:646 10.3389/fpsyg.2014.00646 25018742 PMC4073287

[20] Davidson JW, Howe MJA, Moore DG, Sloboda JA. The role of parental influences in the development of musical performance. Br J Dev Psychol. 1996;14:399–412.

[21] McPherson GE, Zimmermann BJ. Self-regulation of musical learning: A social cognitive perspective In: Colwell R, Richardson C, editors. The new Handbook of Research on Music Teaching and Learning. New York: Oxford University Press; 2002 p. 327–347.

[22] Butkovic A, Ullén F, Mosing MA. Personality related traits as predictors of music practice: Underlying environmental and genetic influences. Pers Individ Dif. 2015;74:133–138.

[23] Mosing MA, Madison G, Pedersen NL, Kuja-Halkola R, Ullén F. Practice does not make perfect: no causal effect of music practice on music ability. Psychol Sci. 2014;25(9):1795–1803. 10.1177/0956797614541990 25079217

[24] Hambrick DZ, Oswald FL, Altmann EM, Meinz EJ, Gobet F, Campitelli G. Deliberate practice: Is that all it takes to become an expert? Intelligence. 2014;45:34–45.

[25] Hambrick DZ, Altmann EM, Oswald FL, Meinz EJ, Gobet F. Facing facts about deliberate practice. Front Psychol. 2014;5. 10.3389/fpsyg.2014.00751 PMC4101876 25101023

[26] Plomin R, Shakeshaft NG, McMillan A, Trzaskowski M. Nature, Nurture, and Expertise. Intelligence. 2014;45:46–59. 24948844 10.1016/j.intell.2013.06.008 PMC4058777

[27] Simonton DK. Exceptional talent and genius In: Chamorro-Tremuzic T, von Stumm S, Furnham A, editors. The Wiley-Blackwell Handbook of Individual Differences. 1st ed Chichester, United Kingdom: Blackwell Publishing Ltd.; 2011 p. 635.

[28] Bunch M, Chapman J. Taxonomy of singers used as subjects in scientific research. J Voice. 2000;14(3):363–369. 11021503 10.1016/s0892-1997(00)80081-8

[29] Moore BC. Frequency difference limens for short-duration tones. J Acoust Soc Am. 1973;54(3):610–619. 4754385 10.1121/1.1913640

[30] Micheyl C, Delhommeau K, Perrot X, Oxenham AJ. Influence of musical and psychoacoustical training on pitch discrimination. Hear Res. 2006;219(1–2):36–47. 16839723 10.1016/j.heares.2006.05.004

[31] Tervaniemi M, Just V, Koelsch S, Widmann A, Schroger E. Pitch discrimination accuracy in musicians vs nonmusicians: an event-related potential and behavioral study. Exp Brain Res. 2005;161(1):1–10. 15551089 10.1007/s00221-004-2044-5

[32] Schellenberg EG, W. Weiss M. Music and Cognitive Abilities In: Deutsch D, editor. The Psychology of Music. 3rd ed: Elsevier; 2013 p. 499–550.

[33] Hutchins SM, Peretz I. A frog in your throat or in your ear? Searching for the causes of poor singing. J Exp Psychol Gen. 2012;141(1):76–97. 10.1037/a0025064 21875245

[34] Russo FA, Thompson WF. An interval size illusion: The influence of timbre on the perceived size of melodic intervals. Percept Psychophys. 2005;67(4):559–568. 16134451 10.3758/bf03193514

[35] Hutchins S, Roquet C, Peretz I. The vocal generosity effect: How bad can your singing be? Music Percept. 2012;30(2):147–159.

[36] Vurma A, Raju M, Kuuda A. Does timbre affect pitch?: Estimations by musicians and non-musicians. Psychol Music. 2010;39(3):291–306.

[37] Zarate JM, Ritson CR, Poeppel D. The effect of instrumental timbre on interval discrimination. PLoS One. 2013;8(9):1–9. 10.1371/journal.pone.0075410 PMC3774646 24066179

[38] Warrier CM, Zatorre RJ. Influence of tonal context and timbral variation on perception of pitch. Percept Psychophys. 2002;64(2):198–207. 12013375 10.3758/bf03195786

[39] Fujioka T, Trainor LJ, Ross B, Kakigi R, Pantev C. Musical training enhances automatic encoding of melodic contour and interval structure. J Cogn Neurosci. 2004;16(6):1010–1021. 15298788 10.1162/0898929041502706

[40] Schön D, Magne C, Besson M. The music of speech: Music training facilitates pitch processing in both music and language. Psychophysiology. 2004;41(3):341–349. 15102118 10.1111/1469-8986.00172.x

[41] Koelsch S, Jentschke S, Sammler D, Mietchen D. Untangling syntactic and sensory processing: an ERP study of music perception. Psychophysiology. 2007;44(3):476–490. 17433099 10.1111/j.1469-8986.2007.00517.x

[42] Schellenberg EG, Moreno S. Music lessons, pitch processing, and g. Psychol Music. 2010:209–221.

[43] Strait DL, Kraus N, Parbery-Clark A, Ashley R. Musical experience shapes top-down auditory mechanisms: Evidence from masking and auditory attention performance. Hear Res. 2010;261(1–2):22–29. 10.1016/j.heares.2009.12.021 20018234

[44] Lee H, Noppeney U. Music expertise shapes audiovisual temporal integration windows for speech, sinewave speech, and music. Front Psychol. 2014;5:868 10.3389/fpsyg.2014.00868 25147539 PMC4124486

[45] Kinney DW. Internal consistency of performance evaluations as a function of music expertise and excerpt familiarity. J Res Music Edu. 2009;56(4):322–337.

[46] Morrison SJ, Montemayer M, Wiltshire ES. The effect of a recorded model on band students’ performance self-evaluations, achievement and attitude. J Res Music Edu. 2004;52:116–129.

[47] Fiske HE. Judge-group differences in the rating of secondary school trumpet performances. J Res Music Edu. 1975;23:186–196.

[48] Fiske HE. Relationship of selected factors in trumpet performance adjudication. J Res Music Edu. 1977;25:256–263.

[49] Fiske HE. The effect of a training procedure in musical performance evaluation on judge reliability. Canada, Toronto: Ontario Educational Research Council Report. 1978.

[50] Wapnick J, Flowers PJ, Alegant M, Jasinskas L. Consistency in piano performance evaluation. J Res Music Edu. 1993(41):282–292.

[51] Besson M, Faïta F. An Event-Related Potential (ERP) study of musical expectancy: Comparison of musicians with nonmusicians. J Exp Psychol Hum Percept Perform. 1995;21(6):1278–1296.

[52] Jonaitis EMM, SJ R.. Learning harmony: The role of serial statistics. Cogn Sci. 2009;33:951–968. 10.1111/j.1551-6709.2009.01036.x 21585492

[53] Loui P, Wessel DL, Kam CLH. Humans rapidly learn grammatical structure in a new musical scale. Music Percept. 2010;27(5):377–388. 20740059 10.1525/mp.2010.27.5.377 PMC2927013

[54] Bigand E, Delbé C. L’apprentissage implicite de la musique occidentale In: Kolinsky R, Morais J, Peretz I, editors. Musique, Langage, Emotion: Approche neuro-cognitive Rennes, France: Presses Universitaires de Rennes; 2010 p. 35–47.

[55] Hannon E, Trainor L. Music acquisition: effects of enculturation and formal training on development. Trends Cogn Sci. 2007. 10.1016/j.tics.2007.08.008 17981074

[56] Müllensiefen D, Gingras B, Musil J, Stewart L. The musicality of non-musicians: an index for assessing musical sophistication in the general population. PLoS One. 2014;9(2):e89642 10.1371/journal.pone.0089642 24586929 PMC3935919

[57] Stalinski SM, Schellenberg EG. Music cognition: a developmental perspective. Top Cogn Sci. 2012;4(4):485–497. 10.1111/j.1756-8765.2012.01217.x 22811391

[58] Trainor LJ. Are there critical periods for musical development? Dev Psychobiol. 2005;46(3):262–278. 15772967 10.1002/dev.20059

[59] Trainor LJ, Marie C, Gerry D, Whiskin E, Unrau A. Becoming musically enculturated: effects of music classes for infants on brain and behavior. Ann N Y Acad Sci. 2012;1252:129–138. 10.1111/j.1749-6632.2012.06462.x 22524350

[60] Marmel F, Tillmann B, Dowling WJ. Tonal expectations influence pitch perception. Percept Psychophys. 2008;70(5):841–852. 18613632 10.3758/pp.70.5.841

[61] Dowling WJ, Fujitani DS. Contour, interval, and pitch recognition in memory for melodies. J Acoust Soc Am. 1971;49(2): 524–531. 5541747 10.1121/1.1912382

[62] Edworthy J. Interval and contour in melody processing. Music Percept. 1985;2:375–388.

[63] Stalinski SM, Schellenberg EG, Trehub SE. Developmental changes in the perception of pitch contour: Distinguishing up from down. J Acoust Soc Am. 2008;124(3):1759 10.1121/1.2956470 19045665

[64] Trainor LJ, Trehub SE. A comparison of infants' and adults' sensitivity to western musical structure. J Exp Psychol Hum Percept Perform. 1992;18(2):394–402. 1593226 10.1037//0096-1523.18.2.394

[65] Bigand E, Poulincharronnat B. Are we “experienced listeners”? A review of the musical capacities that do not depend on formal musical training. Cognition. 2006;100(1):100–130. 16412412 10.1016/j.cognition.2005.11.007

[66] Peretz I, Champod AS, Hyde K. Varieties of Musical Disorders: The Montreal Battery of Evaluation of Amusia. Ann N Y Acad Sci. 2003;999:58–75. 14681118 10.1196/annals.1284.006

[67] Novembre G, Keller PE. A conceptual review on action-perception coupling in the musicians' brain: what is it good for? Front Hum Neurosci. 2014;8:603 10.3389/fnhum.2014.00603 25191246 PMC4139714

[68] Kajihara T, Verdonschot RG, Sparks J, Stewart L. Action-perception coupling in violinists. Front Hum Neurosci. 2013;7:349 10.3389/fnhum.2013.00349 23908612 PMC3726832

[69] Haueisen J, Knosche TR. Involuntary motor activity in pianists evoked by music perception. J Cogn Neurosci. 2001;13(6):786–792. 11564322 10.1162/08989290152541449

[70] Gebel B, Braun C, Kaza E, Altenmüller E, Lotze M. Instrument specific brain activation in sensorimotor and auditory representation in musicians. Neuroimage. 2013;74:37–44. 10.1016/j.neuroimage.2013.02.021 23454048

[71] Lahav A, Saltzman E, Schlaug G. Action representation of sound: audiomotor recognition network while listening to newly acquired actions. J Neurosci. 2007;27(2):308–314. 17215391 10.1523/JNEUROSCI.4822-06.2007 PMC6672064

[72] Walfish S, McAlister B, O'Donnell P, Lambert MJ. An investigation of self-assessment bias in mental health providers. Psychol Rep. 2012;110(2):639–644. 22662416 10.2466/02.07.17.PR0.110.2.639-644

[73] Stalinski SM, Schellenberg EG, Trehub SE. Developmental changes in the perception of pitch contour: distinguishing up from down. J Acoust Soc Am. 2008;124(3):1759–1763. 10.1121/1.2956470 19045665

[74] Pfordresher PQ, Larrouy-Maestri P. On drawing a line through the spectrogram: how do we understand deficits of vocal pitch imitation? Front Hum Neurosci. 2015;9:271 10.3389/fnhum.2015.00271 26029088 PMC4432667

[75] Larrouy-Maestri P, Magis D, Morsomme D. Effects of melody and technique on acoustical and musical features of western operatic singing voices. J Voice. 2014;28(3):332–340. 10.1016/j.jvoice.2013.10.019 24495421

[76] Larrouy-Maestri P, Gosselin L, Blanckaert E, Morsomme D. Listeners’ tolerance when listening to melodic performances. Ninth Triennial of the European Society for the Cognitive Sciences of Music 17–22 August; Manchester, UK2015.

[77] Tsay CJ. Sight over sound in the judgment of music performance. Proc Natl Acad Sci U S A. 2013;110(36):14580–14585. 10.1073/pnas.1221454110 23959902 PMC3767512

